# Large Language Models and the "Centaur Model" in Urological Training in Latin America

**DOI:** 10.1590/S1677-5538.IBJU.2025.0632

**Published:** 2025-12-25

**Authors:** Juan Martín Montoya Osorio

**Affiliations:** 1 Universidad de los Andes Bogotá Colombia Universidad de los Andes, Bogotá, Colombia

To the editor,

I read with great interest the letter by Reis "ChatGPT for medical applications and urological science" (1). The authors highlight the potential of large language models (LLMs) to support clinical reasoning, scientific writing, and knowledge synthesis. Here, I expand this discussion by examining their relevance for urological training in resource-variable environments, particularly in Latin America.

In Colombia and other countries of the region, urology residency programs face persistent structural constraints. Surgical exposure varies considerably across institutions; high- volume centers are geographically concentrated, and access to simulation labs, robotic surgery, and minimally invasive training remains uneven (2). Moreover, many programs operate with limited protected academic time and non-standardized mentorship structures, contributing to variability in operative autonomy and readiness for independent practice among graduating residents.

Within this context, LLM-based tools may offer a standardized cognitive scaffold to help reduce disparities in academic exposure. However, while generative models excel at producing fluent text, their internal reasoning processes are often opaque. This contrasts with explainable AI (XAI), which emphasizes interpretability and traceability of model outputs in clinical contexts (3). For educational purposes, the distinction is crucial: medical training must cultivate clinical judgment, not merely produce correct answers.

Recent studies published in this Journal illustrate both the promise and limitations of these tools. Braga et al. demonstrated that ChatGPT can provide helpful general frameworks for pediatric urology but that its clinical suggestions may sometimes be incomplete or misleading, requiring expert oversight (4). Pinto et al. similarly found that although ChatGPT aligned more closely with guideline-based recommendations in post-prostatectomy incontinence management, both LLMs still required specialist supervision for safe application (5). Together, these findings suggest that LLMs may support cognitive development in training but should not function as autonomous clinical guides.

This aligns with the "centaur model" of medical practice, wherein clinicians and AI systems collaborate, each compensating for the limitations of the other (6). In surgical training, the clinician contributes contextual interpretation, ethical reasoning, and adaptability, while the AI system provides structured analytical support and rapid access to medical evidence. When combined with XAI-based learning interfaces, this hybrid approach may help address academic inequities across training programs.

However, responsible integration remains essential. Challenges include hallucination risk, underrepresentation of Latin American populations in training data, language-adaptation barriers, and the potential to reinforce existing educational disparities. Therefore, LLMs should be incorporated as supervised, curriculum-embedded tools rather than independent instructional or evaluative agents.

Reis LO (1) initiated an important discussion. The next step is to evaluate supervised, context-sensitive LLM-based educational interventions across urology residency programs in low- and middle-income settings, where training variability remains a major structural barrier (2).

The Author

## Data Availability

Uninformed
